# Successful management of chromoblastomycosis utilizing conventional antifungal agents and imiquimod therapy

**DOI:** 10.1186/s12941-024-00718-y

**Published:** 2024-06-20

**Authors:** Jinjin Zheng, Shougang Liu, Zhenmou Xie, Yangxia Chen, Liyan Xi, Hongfang Liu, Yinghui Liu

**Affiliations:** 1 Department of Dermatology, Dermatology Hospital, Southern Medical University, Guangzhou, 510091 China; 2 Department of Dermatology, Sun Yat-Sen Memorial Hospital, Sun Yat-Sen University, Guangzhou, China

**Keywords:** Chromoblastomycosis, Reflectance confocal microscopy, Imiquimod, Treatment

## Abstract

Chromoblastomycosis (CBM), a chronic fungal infection affecting the skin and subcutaneous tissues, is predominantly caused by dematiaceous fungi in tropical and subtropical areas. Characteristically, CBM presents as plaques and nodules, often leading to scarring post-healing. Besides traditional diagnostic methods such as fungal microscopy, culture, and histopathology, dermatoscopy and reflectance confocal microscopy can aid in diagnosis. The treatment of CBM is an extended and protracted process. Imiquimod, acting as an immune response modifier, boosts the host’s immune response against CBM, and controls scar hyperplasia, thereby reducing the treatment duration. We present a case of CBM in Guangdong with characteristic reflectance confocal microscopy manifestations, effectively managed through a combination of itraconazole, terbinafine, and imiquimod, shedding light on novel strategies for managing this challenging condition.

## Introduction

CBM is a chronic fungal infection affecting the skin and subcutaneous tissues, that has seen an increased incidence in tropical regions. It commonly presents as plaques and nodules, often resulting in scarring post-healing. Traditional diagnostic methods for CBM include fungal microscopy, culture, and histopathology, while newer techniques such as dermatoscopy and reflectance confocal microscopy aid in early diagnosis. Classical treatment of CBM involves prolonged oral administration of conventional antifungal drugs such as itraconazole and terbinafine. However, long treatment time, impairment of liver function, and poor curative effect still occur. Imiquimod, an immune response modifier, has shown promise in boosting the host’s immune response against CBM and controlling scar hyperplasia. Here, we report a case of CBM utilizing dermatoscopy and reflectance confocal microscopy as supplementary diagnostic tools. A combination therapy involving itraconazole, terbinafine and imiquimod was administered. This innovative approach showcases the potential of integrating traditional antifungal medications with immunomodulatory agents to improve treatment outcomes in CBM, thereby unveiling novel strategies for addressing this challenging condition.

## Case Report

**Fig. 1:**
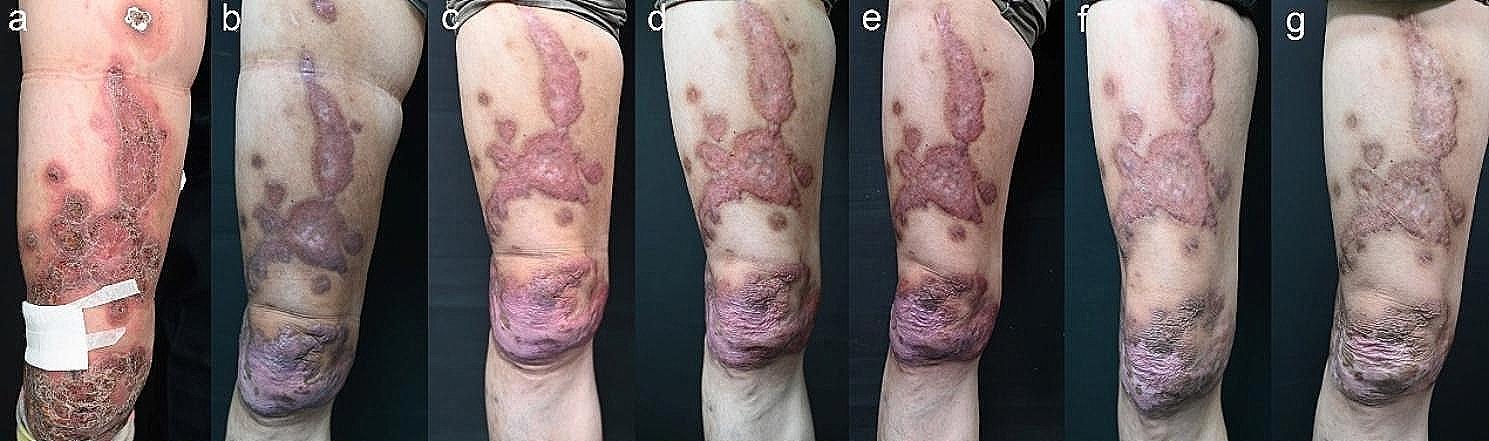
Clinical manifestation: A well-defined dark red plaque on the right leg with scales and crusts on the surface(**a**). The patient had previously been treated with oral itraconazole and terbinafine 3 months and proved subtherapeutic effect(**b** for 1 month and **c** for 3 months). Imiquimod improved the lesions was observed after 1, 2, 3, 5, 10 months (**d**, **e** and **f**, **g** respectively)

The case involved a 55-year-old male farmer with a five-year history of persistent, painful red plaque on his right leg. The lesion had gradually enlarged without any associated trauma. The patient sought evaluation at a local hospital where the exact treatment was unknown and appeared to be ineffective. On physical examination, a well-defined dark red plaque was observed on the right leg, exhibiting scales and crusts on the surface. The surrounding skin appeared red without vesicles, ulcers, or secretions (Fig. 1a).

**Fig. 2:**
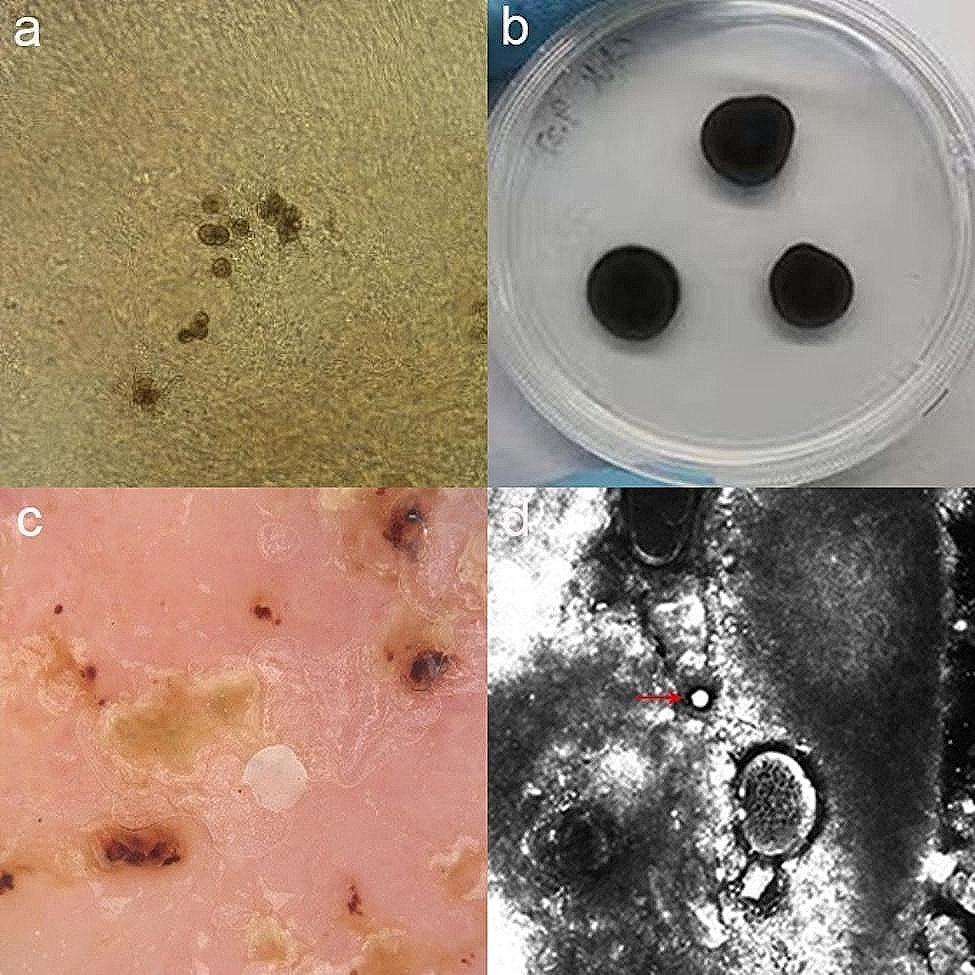
(**a**) Direct microscopic examination from the skin scrapings revealed sclerotic bodies (×400); (**b**) Macroscopic appearance of a *Fonsecaea monophora* colony; (**c**) Dermoscopic finding of the lesions showed small black dots, crusts, scales, and yellow-orange characteristic structures (×10); (**d**) Reflectance confocal microscopy showed small round hyperreflective bodies (red arrow). (basic image 0.5 × 0.5 mm)

Examination of skin scrapings and crushed tissue smears in 10% potassium hydroxide revealed brown cells with irregular septae and thick walls (Fig. 2a). Mycological culture on Sabouraud dextrose agar exhibited the growth of black filamentous colonies after 14 days of incubation at 25 °C (Fig. 2b). The strain was identified as *Fonsecaea monophora* through sequencing of the internal transcribed spacer regions (ITS1 and ITS2) of rDNA, aligning the sequences with those in GenBank using the Basic Local Alignment Search Tool (BLAST). A drug sensitivity test was conducted using a commercial drug sensitivity panel (Yeast One), which included nine antifungal drugs but not terbinafine. A separate drug sensitization panel for terbinafine was self-administered following CLSI M38-A3 guidelines. Itraconazole and terbinafine showed lower minimum inhibitory concentrations compared to fluconazole and amphotericin but higher than voriconazole.

**Fig. 3:**
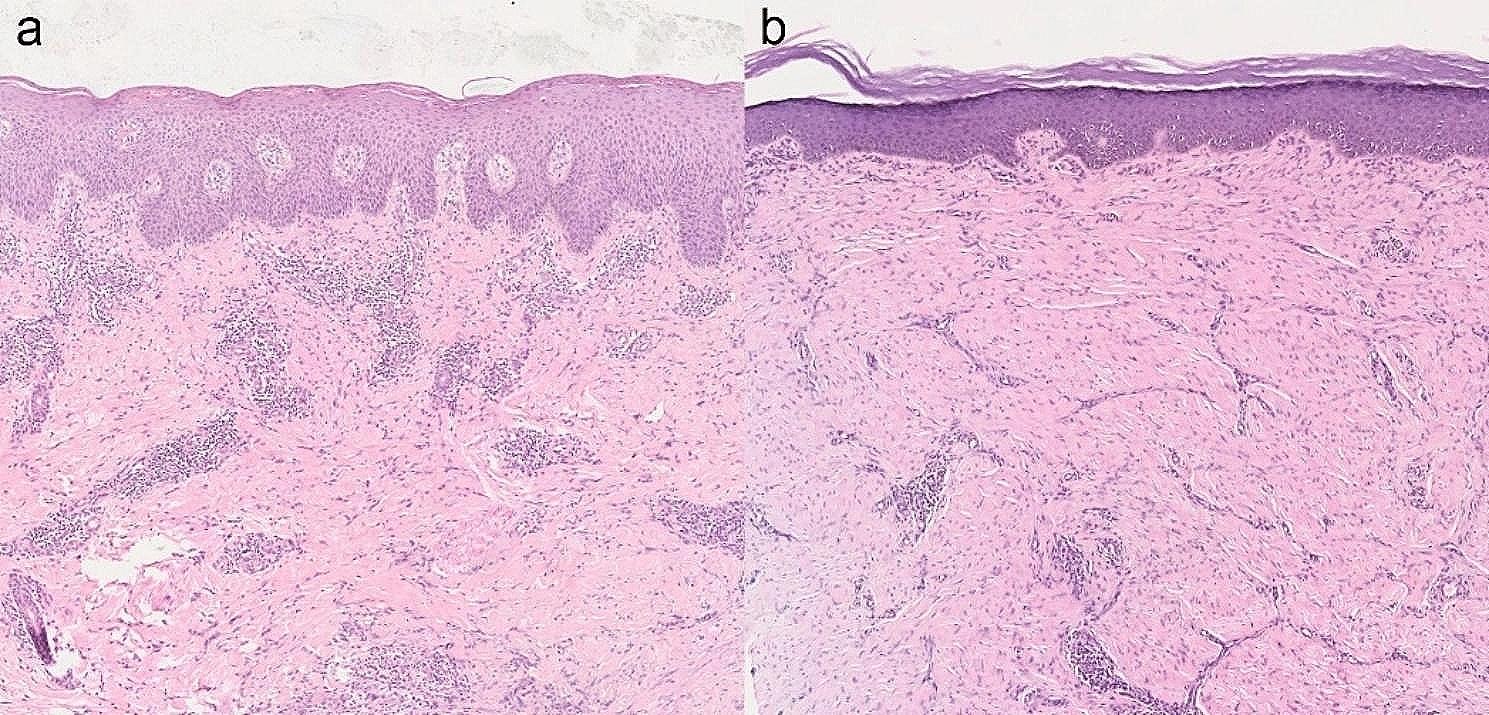
(**a**) Histopathological study revealed an intense inflammatory infiltrate characterized by lymphocytes, eosinophils, and multinucleated giant cells. (**b**) Histopathological study revealed a reduction in inflammatory cells and an increased proportion of scar tissue compared to the initial findings. (HE Staining, ×50)

Dermoscopy revealed small black dots, crusts, scales, and yellow-orange characteristic structures (Fig. 2c). Reflectance confocal microscopy showed small round hyperreflective bodies (Fig. 2d). Histopathological study revealed an intense inflammatory infiltrate characterized by lymphocytes, eosinophils, and multinucleated giant cells (Fig. 3a). No sclerotic body were observed with periodic acid-schiff (PAS) staining. Next-generation sequencing (NGS) of the ground skin tissue showed 93.75% homology (315 bp) with those of *Fonsecaea monophora* (XM_022649996.1).

**Fig. 4:**
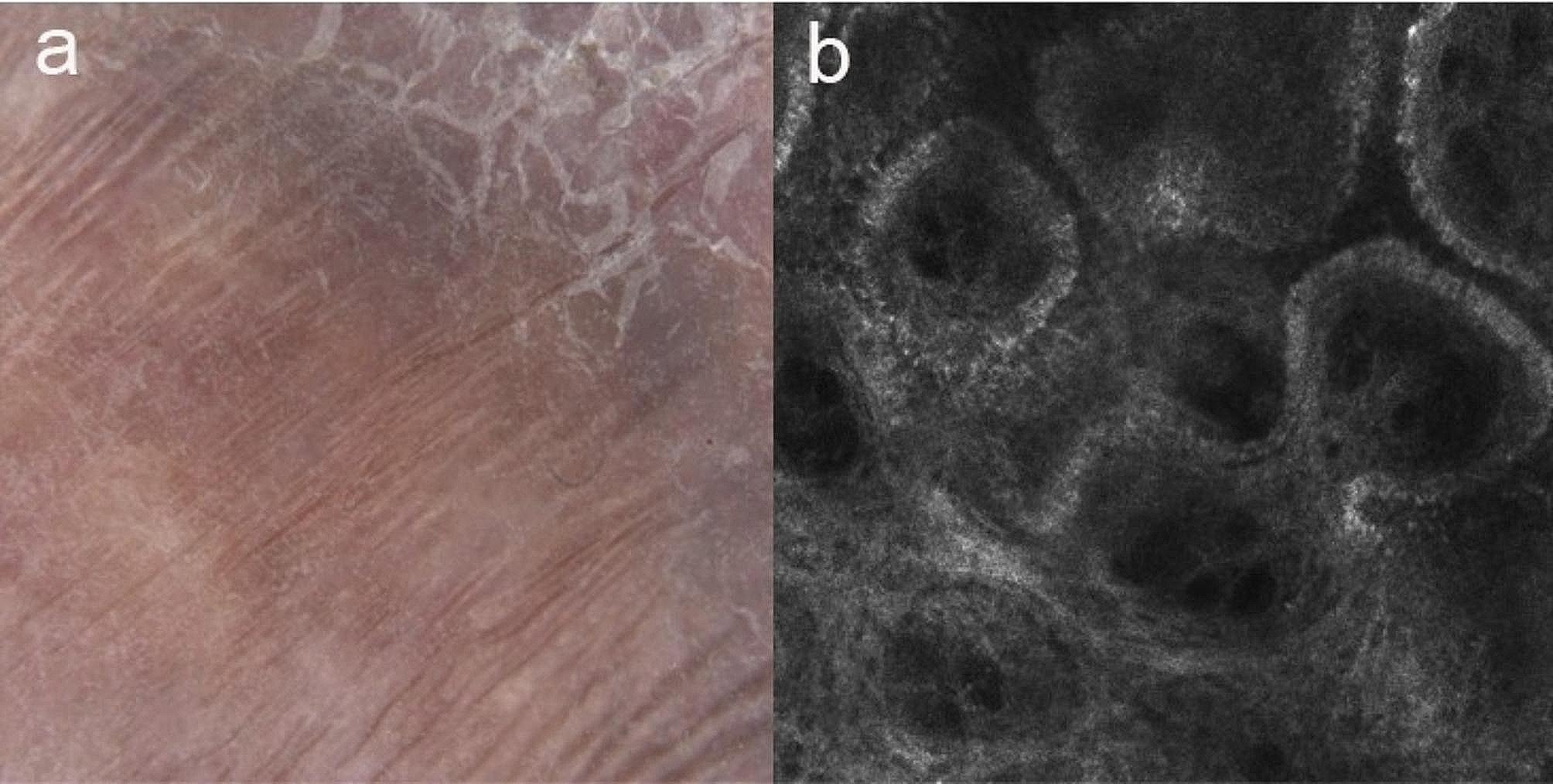
(**a**) After 10 months of treatment, dermoscopic finding of the lesions showed scar and scales, without small black dots, crusts, and yellow-orange characteristic structures (×10); (**b**) No small round hyperreflective bodies seen on reflectance confocal microscopy after 10 months of treatment (basic image 0.5 × 0.5 mm)

The patient had previously been treated with oral itraconazole (200 mg qd) and terbinafine (250 mg qd) for 3 months, which proved subtherapeutic effect (Fig. 1b and c). Subsequently, a combination of topical 5%imiquimod (0.6 g qod) was initiated, showing efficacy after two months of treatment (Fig. 1d-g). Histopathological study demonstrated a reduction in inflammatory cells and an increased proportion of scar tissue compared to the initial findings (Fig. 3b). Dermoscopic finding of the lesions showed scar and scales, without small black dots, crusts, and yellow-orange characteristic structures, No small round hyperreflective bodies seen on reflectance confocal microscopy after 10 months of treatment (Fig. 4). Throughout the treatment period, no itching or burning sensations were reported. Additionally, routine monthly blood tests and assessments of liver and kidney function showed no abnormalities.

## Discussion

The main pathogens of CBM include *Fonsecaea pedrosoi*, *Fonsecaea monophora*, *Fonsecaea nubica*, and *Fonsecaea pugnacious* [1]. *Fonsecaea monophora* has emerged as a significant pathogen in the southern region, responsible for a considerable number of reported cases in Guangdong [2]. Typically, the infection originates from breaches in the skin barrier, such as those caused by plant punctures from thorns or wood, a common occupational hazard in farming [1]. This aligns with the patient profile in this case, where a male farmer, primarily with right lower extremity involvement, presented with plaques, accompanied by crusting, scaling, and erythema. However, the specific history of trauma leading to the infection remained unclear, suggesting the possibility of neglected or unnoticed causative factors.

The current diagnostic approach for CBM involves clinical presentation, mycological examination, and histopathological study. However, these methods often necessitate considerable time. Dermoscopy and reflectance confocal microscopy offer rapid, noninvasive detection of characteristic abnormalities, aiding in early diagnoses and monitoring disease progression. Dermoscopic findings may reveal dark brown to black pitting, crusting, scaling, and orange-yellow discoloration, with multiple irregular reddish-black pits indicating inflammatory cell and fungal element elimination [3–6]. Reflectance confocal microscopy provides real-time images, visualizing sclerotic bodies as bright white spherical vesicles due to melanin presence [7]. These imaging techniques are crucial for early identification and differential diagnosis, especially in atypical CBM cases. While they aid initial diagnosis, confirmation still relies on mycologic examination and histopathologic study. Combining direct microscopy, dermoscopy, and reflectance confocal microscopy facilitates early diagnosis and treatment planning. The patient marks the first reported case in China demonstrating typical reflectance confocal microscopy findings, characterized by small round hyperreflective bodies.

CBM often induces hypertrophic scarring or tissue fibrosis due to the formation of sclerotic bodies, presenting a challenge for drug penetration. CBM might be linked to a defect in innate Toll-like receptor recognition, a deficiency that can be rectified by the exogenous administration of Toll-like receptor agonists [8]. Imiquimod, functioning as a Toll-like receptor agonist, has demonstrated efficacy in treating external anogenital warts, actinic keratoses, and superficial basal cell carcinomas. Studies have indicated its benefits in CBM treatment by modulating immunity and shortening the treatment course [9–19]. In addition, imiquimod inhibits collagen synthesis by promoting the production of the Th1 cytokine TNF-γ, and also inhibits the Th2 cellular immune response by inhibiting the production of the Th2 cytokines IL-4, IL-5, etc., ultimately controlling scar hyperplasia [20, 21]. In this case, conventional antifungal medications alone provided suboptimal results. However, after one month of combining imiquimod with the patient’s treatment regimen, the skin lesions exhibited significant flattening, and histopathology revealed a marked reduction in inflammatory cells. We speculate that imiquimod played a role in enhancing host immune cell function and anti-fibrotic effects during this process, thereby shortening the treatment duration.

## Data Availability

No datasets were generated or analysed during the current study.

## Abbreviations

CMB: Chromoblastomycosis
PAS: Periodic acid-schiff
HE: Hematoxylin and eosin
ITS: Internal transcribed spacer
BLAST: Basic Local Alignment Search Tool
NGS: Next-generation sequencing
